# Accumulation pattern of polycyclic aromatic hydrocarbons using *Plantago lanceolata* L. as passive biomonitor

**DOI:** 10.1007/s11356-021-16141-1

**Published:** 2021-09-02

**Authors:** Katalin Hubai, Nora Kováts, Tsend-Ayush Sainnokhoi, Gábor Teke

**Affiliations:** 1 Centre of Natural Sciences, University of Pannonia, Egyetem str. 10, Veszprém, 8200 Hungary; 2 School of Veterinary Medicine, Mongolian University of Life Sciences, Khan-Uul district, Zaisan, Ulaanbaatar, 17042 Mongolia; 3 ELGOSCAR-2000 Environmental Technology and Water Management Ltd., Balatonfuzfo, 8184 Hungary

**Keywords:** Biomonitoring, *Plantago lanceolata*, Polycyclic aromatic hydrocarbons, Vegetative vigour test, Biomass burning, Diesel exhaust

## Abstract

Biomonitors are considered a cheap alternative of active air samplers, especially where spatial pattern of air quality is to be monitored, requiring numerous parallel measurements. Of higher plants, *Plantago lanceolata* L. has been proven a good monitor species with proper accumulation capacity. While biomonitoring studies are difficult to compare due to inherent errors such as the diverse plant material used in different studies, the No. 227 OECD GUIDELINE FOR THE TESTING OF CHEMICALS: Terrestrial Plant Test: Vegetative Vigour Test provides a tool to test extract of aerosol samples under controlled laboratory conditions. In our study, this guideline was followed to experimentally treat *Plantago* with the aqueous extract of a diesel exhaust sample. Accumulation pattern of polyaromatic hydrocarbons (PAHs) was assessed and compared to samples collected in the field. Unlike most studies reported in the literature, both in the experimentally treated and field *Plantago* samples, high ratio of high molecular weight PAHs was experienced. Distribution pattern of accumulated PAHs showed strong correlation between the experimentally treated sample and most of the field plantain samples, underlying the usefulness of laboratory treatments for bioaccumulation studies.

## Introduction

Polycyclic aromatic hydrocarbons (PAHs) are persistent organic pollutants which occur ubiquitously in environmental media (Edwards 1987). In urban atmosphere, they mainly originate from anthropogenic activities such as vehicle emissions, domestic heating or industrial processes (Manoli et al. 2016). A wide range of PAHs have proven highly carcinogenic or mutagenic, including the group of Car-PAHs: benzo(a)anthracene, chrysene, benzo(b)fluoranthene, benzo(k)fluoranthene, benzo(a)pyrene (BaP), dibenzo(a,h)anthracene, indeno(1,2,3-cd)pyrene and benzo(g,h,i)perylene (reviewed by Srogi 2007). Choochuay et al. (2020a, b) detected high abundance of indeno(1,2,3-cd)pyrene and benzo(g,h,i)perylene as indicators of traffic emissions in ambient air of different regions in Thailand.

The choice of air sampling strategy has a significant influence on the quality of information gained during an air sampling campaign (Schummer et al. 2014). Use of active air samplers is expensive and requires physical installation and energy supply (Augusto et al. 2013). Passive air samplers with polyurethane foam provide a widely used alternative as they are cheaper and easy to handle. As there is no need for electricity supply, they can be used in remote areas (reviewed by Domínguez-Morueco et al. 2017). Some guidelines, however, require that field-deployed passive samplers should be preserved and transported frozen or near frozen as soon as possible which is not always feasible (Donald et al. 2016). Other drawback is the sensitivity to environmental, mostly meteorological conditions (Marć et al. 2015).

Biomonitors can also be a cheap and viable solution especially where spatial pattern of air quality is to be monitored, requiring parallel measurements at numerous sites (Lehndorff and Schwark 2004). Such biomonitors allow rather accurate estimation of air pollution (Abril et al. 2014) and can even be used in cases when contamination level is low (Baldantoni and Alfani 2016). Also, in comparison with active air sampling instruments, they can be used in remote and unaccessible areas (Blasco et al. 2011). In the European Union, Directive 2004/107/EC (2004) has proposed the use of biomonitoring tools to assess the spatial deposition of PAHs (Directive 2004/107/EC). Considering the relatively long exposure period, results also provide a time-averaged data series (Zhao et al. 2018). Biomonitoring also allows long-term studies to be conducted covering even decades (Dołęgowska et al. 2021). Although it is a highly accepted tool for assessing environmental quality, Doucette et al. (2018) conclude in their review that inter-study comparisons would be extremely difficult due to the diversity of experimental approaches. As such, several efforts have been made to standardise experimental protocols (e.g. Weber et al. 2018).

PAHs might occur in the gaseous phase (mainly PAHs with higher vapour pressure) and might also be bound to particles (mainly PAHs with lower vapour pressure). Plants are exposed to both gaseous and particle-bound PAHs. Compounds from the vapour phase can get into the interior of the leaves by stomatal uptake and/or diffusion through wax layers and membranes (Tian et al. 2019). As the high molecular weight (HMW) PAHs are mainly associated with particles, it was supposed that they would remain on the leaf surface (Larsson and Sahlberg 1982). Other studies, however, have since proven that compounds can be desorbed from particles and diffuse into the cuticle (Bakker et al. 2000). PAH compounds accumulate in the lipophilic cuticular wax layer which suggests that accumulation will depend on important leaf characteristics such as structure and thickness of cuticular wax (Li et al. 2017).

Passive biomonitoring of PAHs has had a long history. Urban air quality has been successfully assessed using taxonomically different plant taxa, from mosses to higher plants, including different tree species (Lehndorff and Schwark 2004). In addition to these taxa, another widely used group is lichens which are symbiotic associations between fungi and algae and/or cyanobacteria. They can absorb contaminants such as PAHs from both wet and dry atmospheric depositions due to the absence of a protective cuticle layer (Kodnik et al. 2015).

Of herbaceous plants, *Plantago* sp. (plantains) have been favoured for bioaccumulation studies. These species typically live in disturbed habitats such as urban or roadside biotopes. The species is easy to identify even for non-specialists which is also a critical issue when an ideal biomonitor is selected (Al-Alam et al. 2019). Mandal et al. (2018) suggests that edible/medicinal plants which accumulate PAHs can act as transporters for these compounds. In such terms, *Plantago* also is of key importance as in some European countries it is part of the traditional diet and/or used as a medicinal plant (Guarrera and Savo 2016; Sõukand and Pieroni 2016).

Although *Plantago* have been mostly used for assessing heavy metal contamination via accumulation studies (Nadgórska-Socha et al. 2017; Amato-Lourenco et al. 2020), this taxon is also applicable for assessing airborne PAH pollution. In a comparative study, greater plantain (*Plantago major* L.) accumulated higher concentrations of PAHs than grass species, most probably due to leaf characteristics (Bakker et al. 2000). *Plantago lanceolata* L. (narrowleaf plantain) leaves are relatively rough and mildly hairy which influence the deposition of particles and PM-bound chemicals (Howsam et al. 2000). The plant has elongated (lance-shaped) leaves which might also increase accumulation potential: plants with high surface-to-volume ratio tend to accumulate more organic air pollutants than species with compact leaves (Franzaring and van der Eerden 2000).

In addition to application as a passive monitor, *P. lanceolata* was used in bioindication studies. Morphological parameters (stomatal density, specific leaf area and leaf asymmetry) showed significant changes in polluted sites (Kardel et al. 2010; Velickovic and Perisic 2006). Biochemical markers, such as peroxidase and superoxide-dismutase activity, also suggested the fast reaction of this taxon to air pollution (Gostin et al. 2007).

For bioaccumulation studies, different approaches have been in use. Most reported studies collect field samples and measure the concentration of target compounds (St-Amand et al. 2009). In such cases, native, locally or regionally widespread species are favoured (Baldantoni et al. 2014; Nakazato et al. 2018). These studies, however, have some quality assurance constraints: ecotype, age of test plants are not uniform, also, morphological differences might appear due to the fact that environmental parameters of the habitats differ (De Smedt et al. 2018).

Passive monitors can be used in pot studies where test plants are kept at the sampling sites for a definite period (e.g. Capozzi et al. 2020; Dan-Badjo et al. 2007; van Dijk et al. 2015). Other benefit of pot studies is that uniform plant material can be used, such as the standardised culture of the grass species *Lolium multiflorum* ssp. *italicum* (A. Br.) cv. Lema. This test plant has been widely used both in Europe (Klumpp et al. 2009; Rodriguez et al. 2010) and in sub-tropical regions (Rodriguez et al. 2015).

In addition, bioaccumulation studies can be carried out under controlled conditions where both the composition of the material used for treating the test plants and exposure are known (Slaski et al. 2002). Chen et al. (2019) used an enclosed microcosm system to assess the temperature-dependent uptake and accumulation of PAHs.

In our previous study, the No. 227 OECD GUIDELINE FOR THE TESTING OF CHEMICALS: Terrestrial Plant Test: Vegetative Vigour Test was adapted to assess foliar uptake of PAHs from aqueous extract of an urban aerosol and to determine bioconcentration factor of individual PAHs (Kováts et al. 2013; Teke et al. 2020). The aim of the present study was to compare accumulated PAH profiles in *Plantago* samples collected at different locations and in test plants treated experimentally under laboratory conditions. As diesel exhaust extract was used for treatment, comparison to field samples might provide an additional tool to identify possible pollution sources at each sampling site, in parallel with conventional source appointment methods.

## Material and methods

### Study area

**Fig. 1:**
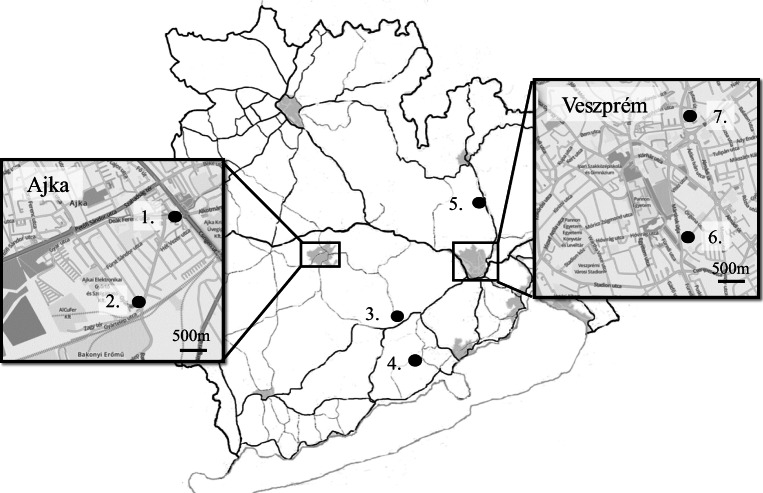
Location of the sampling sites: 1. Ajka Centre; 2. Ajka power plant; 3. Nagyvázsony roadside; 4. Pécsely National Park; 5. Eplény bus stop, 6. Veszprém petrol station; 7. Veszprém bus station

Sampling sites were selected to represent different land use pattern, pollution sources and/or different levels of pollution (Fig 1) with special regard to different levels of traffic. Additional important criteria was the abundance of *P. lanceolata* which had been previously checked. Veszprém is the biggest town in Veszprém county; both sampling sites are situated in the centre, at the proximity of the central bus station and at a major road cutting through the town, near a petrol fuel station. In Ajka, one of the sampling sites is situated close to a thermal power plant which uses biomass (wood); the other sampling site represents the centre of the town with medium traffic. Two other sites (Nagyvázsony and Eplény) are medium-sized settlements. In Nagyvázsony, the sampling spot is situated outskirts of the settlements, at a main road. In Eplény, the sample was taken at a bus stop by a main road, in the centre of the settlement; therefore, this sampling site represents mixed conditions. In order to compare these sites with relatively unpolluted areas, a background location was selected, in the Balaton National Park. This sampling site is not affected by human activities and is situated far from any traffic.

### Plant sampling

At the selected locations, leaves of three to four fully grown plants of *P. lanceolata* were collected. Sampling was carried out in mid June. Leaves were immediately taken to the laboratory, washed with ionic load-free water and frozen (−20 °C) until analysis.

### Experimental treatment

*P. lanceolata* plants were experimentally treated following the No. 227 OECD GUIDELINE FOR THE TESTING OF CHEMICALS: Terrestrial Plant Test: Vegetative Vigour Test.

For treatment, aqueous extract of PM sample was collected from the exhausts of a diesel-powered 13-year-old, Euro4 jeep using a KÁLMÁN PM_2.5_ sampler. Sampling took place in a closed premise about 1 m from the tailpipe; the device was operated at a flow rate of 32 m^3^ h^−1^ for 4 × 10 min at idling. Quartz filters were used (Whatman QMA, diameter 150 mm). Filters were cut in pieces and extracted in 1000 mL high-purity (Milli-Q) water for 24 h. The extract was then filtered on 0.45-μm pore size filter.

Organic *P. lanceolata* seeds were purchased from Szentesimag Ltd. Twenty-five seeds were sown in pots of 15 cm diameter in commercial soil (pH: 6.8 ± 0.5; N (m/m%): min 0.3; P_2_O_5_ (m/m%): min 0.1; K_2_O (m/m%): min 0.3). For the test, 3 uniform plantlets were kept in each pot. Cultivation of the test plants and further testing were conducted in a glass-house; environmental conditions were set following the prescriptions of the guideline (temperature: 22 °C ± 10 °C; humidity: 70% ± 25%; photoperiod: minimum 16 h light; light intensity: 350 ± 50 μE/m^2^/s).

Treatment was applied by spraying the sample on the surface of test plants; exposure started when the plants reached the 4-true leaf stage. In contrary to the guideline which recommends only one spraying at the beginning of the exposure, three treatments were applied as follows: first treatment on day 0, followed by a second treatment 1 week later, on day 7, and then by the third treatment on day 14. The test was terminated on day 21. After exposure, leaves were immediately taken to the laboratory, washed with ionic load-free water and frozen (−20 °C) until analysis.

A control was also set, where plants received foliar spraying with tap water on the same day as treated plants. Both the control and the treated plant series included 10-10 replicates (10-10 pots).

### Determination of the PAH content

Gas chromatography (GC-MS) method has become the preferred method for polycyclic aromatic hydrocarbon analysis in diverse environmental compartments (Choochuay et al. 2020a, b; Deelaman et al. 2020a, b) due to its selectivity and resolution (Poster et al. 2006). In aerosol extract, the PAH concentrations were measured by gas chromatographic-mass spectrometry according to MSZ 1484-6:2003 standard (MSZ 1484-6:2003: Testing of waters. Determination of polycyclic aromatic hydrocarbons (PAH) content by gas chromatographic-mass spectrometry, LOD: 0.005 μg/L per component). The concentration of accumulated PAHs in plant samples were analysed by Agilent 6890GC 5973E MSD GC-MS based on MSZ EN 15527:2009 (Characterization of waste. Determination of polycyclic aromatic hydrocarbons (PAH) in waste using gas chromatography mass spectrometry (GC/MS), LOD: 0.1 μg/kg per component).

Ten grams of plantain leaves was grinded and pounded with 10-g anhydrous sodium sulphate in a ceramic mortar to achieve a homogeneous and representative plant sample. Ten grams of the samples was extracted 3 times with ultrasonic extraction for 20 min with 20 mL n-hexane. Prior to extraction 10 mL acetone was added and the samples were spiked with 100 μL of 0.01 μg/mL deuterated PAH surrogate mixture containing naphtalene-d8, acenaphthene-d10, phenanthrene-d10, chryzene-d12, benzo(a)pyrene-d12 and perylene-d12 (Restek Corporation, Bellefonte, PA, USA).

After the extraction, the sample extract was concentrated in a dry nitrogen stream to 1 mL. With each sample, an additional solid phase silica gel and alumina oxide sample clean-up was performed. The 19 PAHs analysed in this study were as follows: naphthalene (Nap), 2-methyl-naphthalene (Methy-Nap), 1-methyl-naphthalene (Me-Nap), acenaphthylene (Acy), acenaphthene (Ace), fluorene (Flo), phenanthrene (Phe), anthracene (Ant), fluoranthene (Flt), pyrene (Pyr), benzo(a)anthracene (BaA), chrysene (Cry), benzo(b)fluoranthene (BbF), benzo(k)fluoranthene (BkF), benzo(a)pyrene (BaP), benzo(e)pyrene (BeP), indeno(l,2,3-cd)pyrene (Ind), dibenzo(a,h)anthracene (DahA) and benzo(g,h,i)perylene (BghiP).

### GC-MS analysis

For GC-MS measurements, an HP-6890 gas chromatograph was coupled to an HP-5973 (Agilent Technologies, Palo-Alto, USA) quadrupole mass spectrometer (low-resolution single MS). Injector and transfer-line temperatures were 320 °C and 250 °C and source and analyser temperatures were 280 °C and 150 °C. A glass insert, 4 mm i.d., loosely filled with silanized glass wool was used in the split/splitless GC injector (320 °C, purge splitless 1.5 min). The column head pressure was 50 PSI. The GC column was 30 m × 0.25 mm i.d., film thickness 0.25 μm, ZB-Semivolatiles (Phenomenex, Torrance, CA, USA). The GC oven temperature was maintained at 40 °C for 3 min after injection then programmed at 40 °C/min to 80 °C for 0.5 min and increased with 15 °C/min with small break (at 240 °C 8 min) to 310 °C. Helium (N55, Linde, Dublin, Ireland) was used as carrier gas; the constant flow rate of carrier gas was 1.2 mL/min. The acquisition mode was SIM (single ion monitoring). The MSD was scanned from 50 to 550 amu. Electron ionisation was used with energy of 70 eV. A five-point calibration was detected over a concentration range of 0.5–5.0 μg/L for each of the target chemical compound from a standard mixture was established. All target PAHs had good linearity with *R*^2^ values >0.98. The mean recovery based on the extraction of certified standard solution was in the range of 60–120% that demonstrated the good reliability and correctness of the used method. All data were corrected for the average value of the blanks. The limit of PAH detection (LOD) in extract was 0.001 μg/L and in plant samples 0.1 μg/kg dry plant material.

Quality assurance/quality control (QA/QC) internal and surrogate standards were used for quantification and quantifying of sample and for procedural recovery. Internal standard (p-terphenyl-d14, 2-fluorobiphenyl from Restek Corporation, Bellefonte, PA, USA) and surrogate standards (naphtalene-d8, acenaphthene-d10, phenanthrene-d10, chryzene-d12, benzo(a)pyrene-d12 and perylene-d12, from Restek Corporation, Bellefonte, PA, USA) were used. The standards were properly diluted with GC grade solvents (Sigma-Aldrich, St. Louis, MO, USA) and prepared freshly before the analysis. The regulatory requirements of the USA-EPA and EU achieved good recoveries for the compounds ranging 73.5–119.4%. The recoveries were 96–104% for 2-fluorobiphenyl and 108–114% for p-terphenyl-d14. The recoveries of surrogate standards were acceptable for the standards (naphtalene-d8, acenaphthene-d10 82–102%, phenanthrene-d10 92–109%, chrysene-d12 95–107%, perylene-D12 82–91%), which were good for making results reliable.

Analytical determinations were performed by courtesy of the Laboratory of the ELGOSCAR-2000 Environmental Technology and Water Management Ltd. accredited by the National Accreditation Authority (complies with criteria of Standard MSZ EN ISO/IEC 17025:2018), registration number NAH-1-1278/2015.

### Statistical analysis

In order to examine compositional differences among samples, principal component analysis (PCA) has been performed which generally reduces the set of variables into two major principle components. PCA has been extensively used to evaluate PAH accumulation pattern in different plant matrices (e.g. Kodnik et al. 2015; Capozzi et al. 2017).

.Statistical analyses were performed using the RStudio (RStudio Desktop 1.4.1106) programme, ggfortify package (https://CRAN.R-project.org/package=ggfortify) and R 4.0.0 programme (http://cran.r-project.org/src/base/R-4/R-4.0.0.tar.gz) Rcmdr package. Accumulated amounts of PAHs in plants were compared using Spearman correlation; coefficients were determined to estimate the dependence of sampling site on the levels of PAHs found in *Plantago* samples. To identify the relationship between the PAH content of samples and sampling sites, PCA and factor analysis were performed with RStudio. Statistical significance was defined as *p* ≤ 0.05.

## Result and discussion

### PAH profiles in the diesel extract and in the experimentally treated *P. lanceolata* leaves

In the diesel exhaust extract, Phe and Flt were the dominant PAHs, similarly to the study of de Souza and Corrêa (2016) (Table 1). In addition to Phe and Flt, 4-ring Pyr was found dominant in the study of Corrêa et al. (2021). Dominance of these PAHs was reported in other studies as well (Fabiańska et al. 2016; Lin et al. 2019). Most studies agree that in diesel exhaust, ratio of five- or more ring species is very low if any (Jin et al. 2014; Yilmaz and Davis 2016).

**Table 1:** Concentration of PAHs in the aerosol extract and in the experimentally treated plant samples. Bioconcentration factors (BCFs) and molecular weights are also shown. Car PAHs are given in italic

PAH	Diesel extract	*Plantago* treated	BCF	Molecular weight
	(μg/L)	(y)		(g/mol)
Acenaphthylene	0.022	0.53	24.1	152.19
Fluorene	0.034	1.27	37.4	166.22
Phenanthrene	0.264	8.64	32.7	178.23
Anthracene	0.018	0.43	23.9	178.23
Fluoranthene	0.22	4.08	18.5	202.25
Pyrene	0.121	4.19	34.6	202.25
*Benzo(a)anthracene*	0.019	2.9	152.6	228.29
*Chrysene*	0.032	4.13	129.1	228.3
*Benzo(b)fluoranthene*	0.036	15.7	436.1	252.31
*Benzo(k)fluoranthene*	0.013	6.38	490.8	252.31
Benzo(e)pyrene	0.018	8.24	457.8	252.32
*Benzo(a)pyrene*	0.007	13.8	1971.4	252.32
*Indeno(1,2,3-cd)pyrene*	0.008	7.09	886.3	276.33
*Dibenzo(a,h)anthracene*	0.001	2.66	2660.0	278.35
*Benzo(g,h,i)perylene*	0.005	6.36	1272.0	276.3
Total PAH	0.818	92.2	112.7	–

In the leaves of experimentally treated *P. lanceolata* plants, total PAH concentration was 92.2 μg/kg. BbF represented 17% of total individual PAHs, followed by BaP (15%), Phe and BeP (9%), Ind (8%), BkF and BghiP (7%), Pyr and Cry (4.5%), Flt (4.4%) and Nap, BaA and DahA (3%). In general, 5-ring PAHs were dominant, amounting to 50% of total PAHs.

In order to quantify accumulation pattern of individual PAHs, bioconcentration factors were calculated (Table 1). BCF is the substance partition coefficient between the organism and the external medium (Paraíba et al. 2010), in our case the sample used for treatment. BCF was calculated as follows: BCF = PAH concentration in the *P. lanceolata* leaves/PAH concentration in the sample (Kacálková and Tlustoš 2011).

We observed that car PAHs had higher BCF than other individual PAHs; BCF of DahA was 2660, BaP 1971 and Ind 886. Lowest BCF was found in case of Flt (18.5) and Ant (23.9), respectively.

### PAH concentrations in *P. lanceolata* samples collected at different locations

Concentrations of 19 individual PAHs accumulated in *P. lanceolata* were measured (see Table 2). Considering field samples, highest total PAH concentration was found in Veszprém bus station (768 μg/kg), followed by Eplény (70.6 μg/kg), Nagyvázsony (55.8 μg/kg) and Ajka Centre (42.5 μg/kg) while lowest concentrations were observed in Ajka power plant (38.6 μg/kg) and Veszprém petrol station (34.7 μg/kg).

**Table 2:** Concentrations of individual PAHs in different villages (abbreviations, number of rings and molecular weight)

PAHs	Abbreviation	No. of rings	Ajka power plant	Ajka Centre	Veszprém petrol station	Veszprém central bus station	Nagyvázsony	Eplény	Mean (range)	Detection rate
Naphthalene	Nap	2	2.72	4.72	ND	1.93	4.6	2.22	2.7 (0–4.72)	83.3
2-Methyl-naphthalene	Methy-Nap	2	2.01	2.58	1.04	2.21	2.52	1.76	2 (1.04–2.58)	100
1-Methyl-naphthalene	Me-Nap	2	1.15	2.64	1	2.44	1.93	1.05	1.7 (1–2.64)	100
Acenaphthylene	Acl	3	1	0.94	0.82	1.17	0.69	0.9	0.9 (0.69–1.17)	100
Acenaphthene	Ace	3	0.48	0.67	0.34	0.64	0.47	0.51	0.5 (0.34–0.67)	100
Fluorene	Flu	3	1.16	1.41	0.76	1.31	2.01	0.76	1.2 (0.76–2.01)	100
Phenanthrene	Phe	3	6.28	6.89	3.14	8.9	8.48	7.33	6.8 (3.14–8.9)	100
Anthracene	Ant	3	0.22	0.48	0.3	2.03	0.41	0.52	0.6 (0.22–2.03)	100
Fluoranthene	Flt	4	2.26	3.36	2.2	38.4	2.03	10.3	9.76 (2.03–38.4)	100
Pyrene	Pyr	4	1.97	3.47	2.58	72.3	2.43	7.89	15.1 (1.97–72.3)	100
Benzo(a)anthracene	BaA	4	0.7	1.08	1.05	65.5	2.19	3.38	12.3 (0.7–65.5)	100
Chrysene	Cry	4	1.22	1.04	1.06	49.7	1.84	3.28	9.6 (1.04–49.7)	100
Benzo(b)fluoranthene	BbF	5	4.23	3.62	3.15	151	5.89	8.54	29.4 (3.15–151)	100
Benzo(k)fluoranthene	BkF	5	2.81	2.01	1.62	44	2.24	3.24	9.3 (1.62–44)	100
Benzo(e)pyrene	BeP	5	2.2	2.27	1.52	84.4	2.47	3.58	16 (1.52–84.4)	100
Benzo(a)pyrene	BaP	5	3.65	2.66	6.43	88.2	7.7	6.91	19.2 (2.66–88.2)	100
Dibenzo(a,h)anthracene	DahA	5	0.47	ND	0.97	16.1	ND	1.06	4.6 (0.47–16.1)	66.7
Indeno(1.2.3-cd)pyrene	Ind	6	2.02	1.51	3.68	51.4	4.54	4.07	11.2 (1.51–51.4)	100
Benzo(g,h,i)perylene	BghiP	6	2.07	1.23	3.09	85.6	3.41	3.36	16.4 (1.23–85.6)	100
Total PAHs			38.6	42.5	34.7	768	55.8	70.6		

*ND*, not detected

In the background site, all PAHs were under the detection limit.

**Fig. 2:**
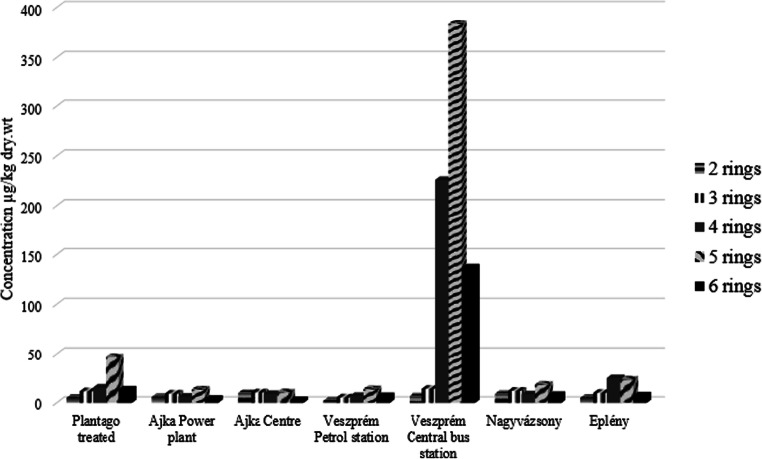
Total amount of different molecular weight PAHs on the sampling spots in the test plants

Figure 2 illustrates the total amount of different molecular weight PAHs. In comparison to the other sampling sites, concentration of HMW PAHs accumulated in *P. lanceolata* was significantly higher in Veszprém central bus station, showing relatively high level of pollution.

The results indicated that most abundant individual PAHs accumulated in *P. lanceolata* were Phe and BbF in all sampling sites except Veszprém petrol station and Eplény. Concentration of Phe (3-ring) was 8.48 μg/kg dry-wt in Nagyvázsony, 6.89 μg/kg dry-wt in Ajka Centre and 6.28 μg/kg dry-wt in Ajka power plant, while concentration of BbF (5-ring) from Veszprém central bus station, *Plantago* treated and Ajka power plant were 151 μg/kg dry-wt, 15.7 μg/kg dry-wt and 10.4 μg/kg dry-wt.

### Comparison of experimentally treated plants and field collections

Distribution pattern of accumulated PAHs in the experimentally treated sample showed strong correlation with the collected *Plantago* samples (Table 3), except for Ajka Centre. Also, strong correlations were found between the different sites, except for Ajka Centre/Veszprém petrol station and Nagyvázsony/Veszprém bus station.

**Table 3:** Correlation between the collected *Plantago* samples

	Ajka Centre	Ajka power plant	Eplény	Nagyvázsony	*Plantago* treated	Veszprém petrol station	Veszprém central bus station
Ajka Centre	–	*t* = 6.9656; df = 17; *p* = 2.28 * 10^−6^; cor = 0.8605	*t* = 3.7369; df = 17; *p* = 0.0016; cor = 0.6716	*t* = 8.8102; df = 17; *p* = 9.582 * 10^−8^; cor = 0.9057	*t* = 4.524; df = 17; *p* = 0.0003; cor = 0.7391	*t* = 3.1421; df = 17; *p* = 0.0059; cor = 0.6061	*t* = 1.5791; df = 17; *p* = 0.1327; cor = 0.3577
Ajka power plant		–	*t* = 3.1818; df = 17; *p* = 0.00545; cor = 0.6109	*t* = 4.6852; df = 17; *p* = 0.0002128; cor = 0.7507	*t* = 1.8749; df = 17; *p* = 0.07809; cor = 0.4139	*t* = 1.3347; df = 17; *p* = 0.1996; cor = 0.3080	*t* = 0.36962; df = 17; *p* = 0.7162; cor = 0.0893
Eplény			–	*t* = 3.0815; df = 17; *p* = 0.006767; cor = 0.5987	*t* = 3.8569; df = 17; *p* = 0.001265; cor = 0.6831	*t* = 3.6883; df = 17; *p* = 0.00182; cor = 0.6667	*t* = 3.2482; df = 17; *p* = 0.0047; cor = 0.6188
Nagyvázsony				–	*t* = 5.0545; df = 17; *p* = 9.778 * 10^−5^; cor = 0.7749	*t* = 4.5884; df = 17; *p* = 0.000261; cor = 0.7438	*t* = 1.8438; df = 17; *p* = 0.0827; cor = 0.4082
*Plantago* treated					–	*t* = 5.6699; df = 17; *p* = 2.768 * 10^−5^; cor = 0.8088	*t* = 5.9182; df = 17; *p* = 0.0000168; cor = 0.8205
Veszprém petrol station	–	–	–	–	–	–	*t* = 3.2381; df = 17; *p* = 0.004834; cor = 0.6176462
Veszprém central bus station	–	–	–	–	–	–	–

**Fig. 3:**
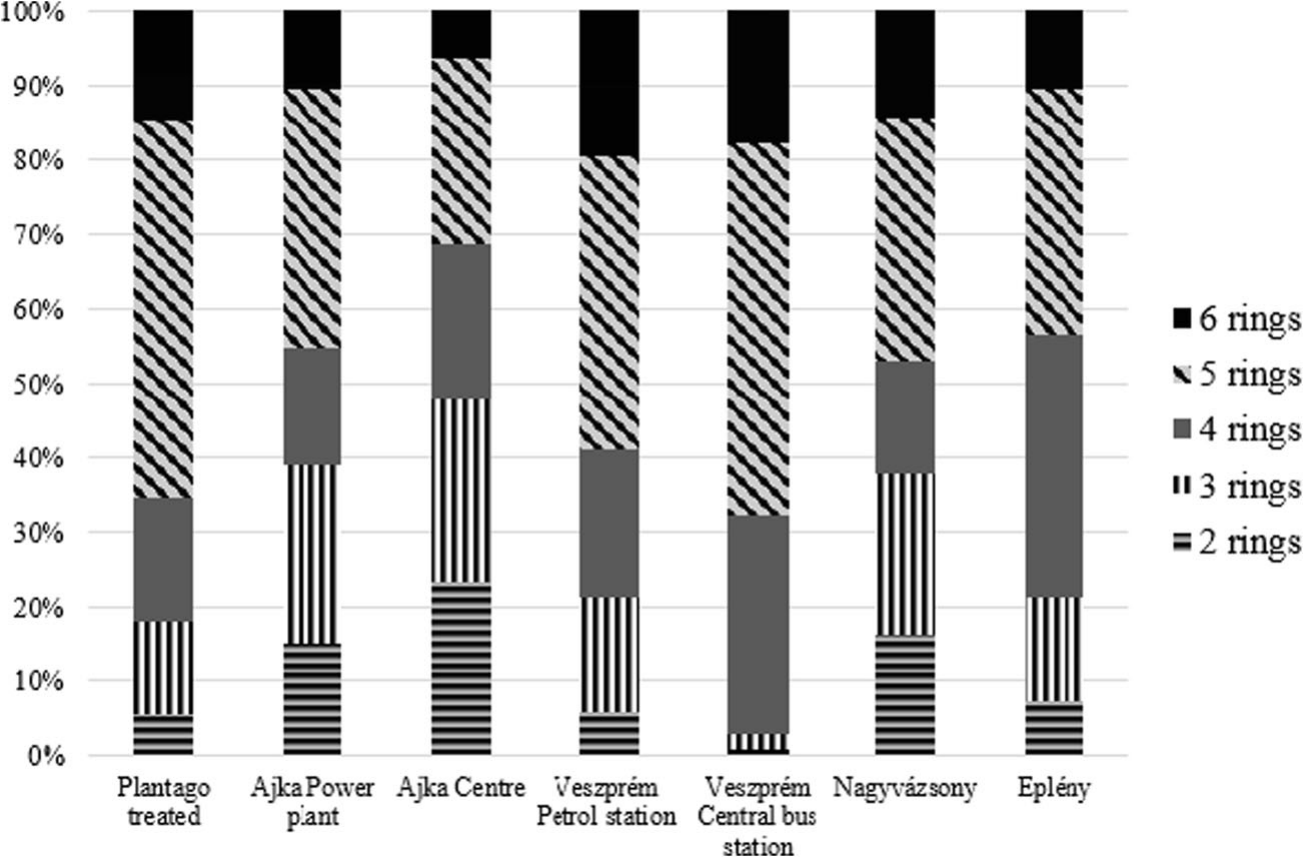
Percentage contribution of different molecular weight PAHs on the sampling spots in the test plants

Both in the experimentally treated and field *Plantago* samples, high ratio of HMV PAHs was experienced (Fig. 3). The results indicated that 5-ring PAHs represented 50% of total PAHs in Veszprém bus station and *Plantago* treated, followed by Veszprém petrol station (39.5%), Ajka Centre (34.6%), Eplény centre (33%), Nagyvázsony roadside (32.8%) and Ajka power plant (24.8%), respectively. Six-ring PAHs occurred in all samples and accounted for 6 to 20% of total PAHs.

In fact, this finding is in contrary with the majority of data reported. In our previous study, lettuce (*Lactuca sativa* L.) was treated with urban aerosol extract and the lower molecular weight (LMW) PAH compounds were predominant after the treatment; Nap and Ant had the highest BCF (Teke et al. 2020). Field studies also report the prevalence of LMW PAHs (e.g. An et al. 2017; Wang et al. 2017; Jia et al. 2018).

However, accumulation pattern might highly depend on the taxon in question (Sæbø et al. 2012). Huang et al. (2018) found that oak leaves show higher accumulating tendency for light and medium molecular weight PAHs in contrary to mosses where stronger accumulating tendency for heavy molecular weight PAHs was experienced. Relatively high share of 5- and 6-ring PAHs was found in grass in the study of Borgulat and Staszewski (2018). Ashraf and Salam (2012) detected elevated concentrations of DahA and BghiP in sampled vegetables such as cabbage. Using *Plantago* in a transplanted pot study, Bakker et al. (1999) also experienced the abundance of heavy molecular weight PAHs.

### Comparison of sampling sites

**Fig. 4:**
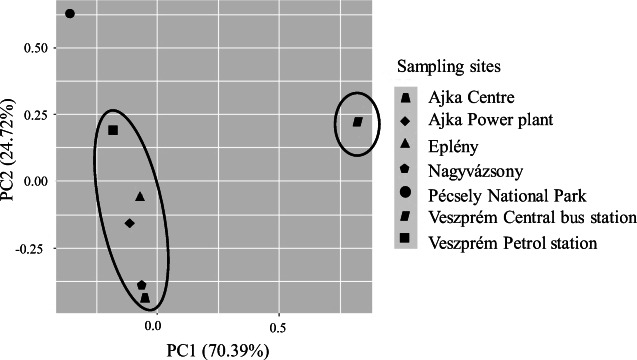
Principal component analysis (PCA) biplot based on the detected PAH concentrations from the sampling spots in the test plants

Principal component analysis (PCA) was used to reduce the number of variables to two principal components (PC1 and PC2) and to establish the relationship between 19 PAHs in *Plantago* samples. The biplot of PCA is presented in Fig. 4. The PC1 component accounted for 68.09% of the total variance and the PC2 component accounted for 24.61% of the total variance. The first principal component (PC1) is in general associated with these 12 PAHs (Ant, Flt, Pyr, BaA, Cry, BbF, BkF, BeP, BaP, DahA, Ind, BghiP) while 7 components (Nap, Flu, Me-Nap, Methy-Nap, Acl, Ace, Phe) from Σ19PAHs correlated with PC2.

The main sources of PAHs correlating with PC1 are markers of coal combustion (Pyr, BaA and Cry) and markers of vehicle (gasoline and diesel) emissions (BkF, BbF and BaP) or coal combustion and vehicle emission markers (Flt, Ant) (Limu et al. 2013). The PAHs correlating with PC2 (Acl, Ace, Flu, Phe, Ant) suggest substantial contribution from low-temperature pyrolysis processes (like biomass combustion or coal combustion) (Yadav et al. 2020).

Two sites are clearly separated as follows: Pécsely National Park which is in fact served as a background sampling site and Veszprém central bus station. In this station, high environmental load is caused by the dominance of diesel-powered, relatively old buses belonging to Euro0–Euro3 European emission standards (Kováts et al. 2013).

**Fig. 5:**
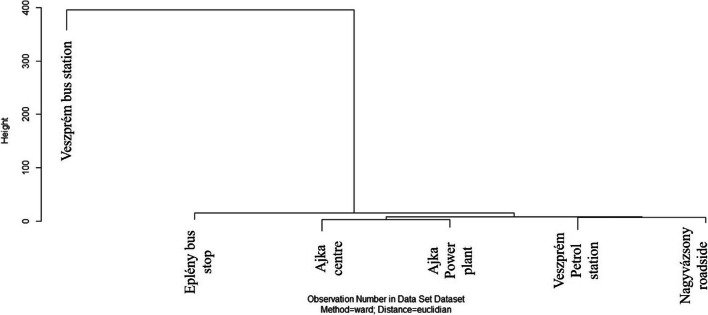
Hierarchical clustering results (HCA) of the PAH concentrations in *Plantago* samples collected in different sampling points. Cluster method: Ward, measure: Euclidean distance

Hierarchical cluster analysis clearly revealed the presence of four clusters (Fig. 5). Veszprém bus station also forms a distinct group, similarly to the results of the PCA. The background sampling site (national park) was not included in this analysis. Considering the rest of the sites, contribution of biomass burning is obvious in case of Ajka power plant, as it uses app. 192.000 tons of wood/year (Gyulai 2006). However, it is interesting to note that in addition to traffic, biomass combustion still provides a significant source despite the fact that sampling was done in June, well after the heating season.

### Limitations of the study

The main aim of the study was to introduce a quasi-standard protocol for lab-scale bioaccumulation tests and to compare the bioaccumulation pattern with actual data. Although PAH concentrations and accumulation pattern gave an easy-to-interpret dataset, some limitations need to be taken into consideration. The most important factor is the age of plant material used as concentration of accumulated pollutants would depend on phenophase/degree of development of the bioaccumulator plant (Baldantoni and Alfani 2016). In addition, in this comparative study, different exposure regimes have been encountered, during the test a fixed but relatively short (21 days) exposure was used. On the other hand, exposure in case of field collected plantains is not known although it is also an important factor influencing the amount of accumulated compounds (Świerk and Szpakowska 2011). However, the fact that concentration of accumulated PAHs in the experimentally treated plants showed good correlation with field samples might indicate the applicability of the protocol for bioaccumulation studies.

## Conclusions

Accumulation of PAHs was compared in experimentally treated *Plantago* leaves and samples collected at different locations. Considering PAH isomers, distribution pattern of accumulated PAHs showed strong correlation between the experimentally treated sample and most of the field plantain samples. Every sample was characterised by the prevalence of HMW PAHs, in contrary with most of the reported studies. The study has shown that *P. lanceolata* is a reliable passive monitor when distribution pattern of PAH contamination is to be assessed. Also, experimental treatment under laboratory conditions provided a comparable reference to field collected samples.

## Data Availability

All data generated or analysed during this study are included in this published article.
